# Using an intervention mapping approach to develop a discharge protocol for intensive care patients

**DOI:** 10.1186/s12913-017-2782-2

**Published:** 2017-12-19

**Authors:** Margo van Mol, Marjan Nijkamp, Christine Markham, Erwin Ista

**Affiliations:** 1 Department of Intensive Care Adults, Erasmus MC University Medical Center, P.O. Box 2040, Room 1005, 3000 CA Rotterdam, The Netherlands; 2 Faculty of Psychology and Educational Sciences, Open University of the Netherlands, Heerlen, The Netherlands; 3 Center for Health Promotion and Prevention Research, University of Texas Health Science Center at Houston, School of Public Health, Houston, USA; 4 Department of Intensive Care Children, Erasmus MC University Medical Center - Sophia Children’s Hospital, Rotterdam, The Netherlands

**Keywords:** Discharge protocol, Emotional distress, Health service research, Intensive care unit, Intervention mapping, Transition procedure

## Abstract

**Background:**

Admission into an intensive care unit (ICU) may result in long-term physical, cognitive, and emotional consequences for patients and their relatives. The care of the critically ill patient does not end upon ICU discharge; therefore, integrated and ongoing care during and after transition to the follow-up ward is pivotal. This study described the development of an intervention that responds to this need.

**Methods:**

Intervention Mapping (IM), a six-step theory- and evidence-based approach, was used to guide intervention development. The first step, a problem analysis, comprised a literature review, six semi-structured telephone interviews with former ICU-patients and their relatives, and seven qualitative roundtable meetings for all eligible nurses (i.e., 135 specialized and 105 general ward nurses). Performance and change objectives were formulated in step two. In step three, theory-based methods and practical applications were selected and directed at the desired behaviors and the identified barriers. Step four designed a revised discharge protocol taking into account existing interventions. Adoption, implementation and evaluation of the new discharge protocol (IM steps five and six) are in progress and were not included in this study.

**Results:**

Four former ICU patients and two relatives underlined the importance of the need for effective discharge information and supportive written material. They also reported a lack of knowledge regarding the consequences of ICU admission. 42 ICU and 19 general ward nurses identified benefits and barriers regarding discharge procedures using three vignettes framed by literature. Some discrepancies were found. For example, ICU nurses were skeptical about the impact of writing a lay summary despite extensive evidence of the known benefits for the patients. ICU nurses anticipated having insufficient skills, not knowing the patient well enough, and fearing legal consequences of their writings. The intervention was designed to target the knowledge, attitudes, self-efficacy, and perceived social influence. Building upon IM steps one to three, a concept discharge protocol was developed that is relevant and feasible within current daily practice.

**Conclusion:**

Intervention mapping provided a comprehensive framework to improve ICU discharge by guiding the development process of a theory- and empirically-based discharge protocol that is robust and useful in practice.

**Electronic supplementary material:**

The online version of this article (10.1186/s12913-017-2782-2) contains supplementary material, which is available to authorized users.

## Background

Admission into an intensive care unit (ICU) is associated with short and long-term physical impairment, cognitive deterioration, and emotional consequences for patients and their relatives [1–4]. Less than 10% of patients who were mechanically ventilated for more than four days are alive and fully independent one year later [5]. In addition to the challenges of recovering from both an underlying disease and physical revalidation, emotional distress post ICU admission needs to be addressed. From USA studies, it is known that 12% to 43% of recovering ICU patients still suffer from some form of anxiety (including paranoia, nightmares, and hallucinations), depression (10 to 30%), or even post-traumatic stress (10% to 64%) [6–8]. In South-Africa, one study showed 58% anxiety, 28% depression, and 32% of post-traumatic stress in post ICU patients [9]. A Chinese study reported a high incidence rate of anxiety (59.7%) in ICU patients’ family members who acted as representatives of the patient, and concluded that nurses should pay more attention to the family members and provide more psychological nursing when taking care of patients [10]. Recently, a Post Intensive Care Syndrome (PICS) has been defined to underscore the total impact of an ICU admission: “The new or worsening impairments in health status arising and the persistence after hospitalization for a critical illness” [5, 11]. These continuing problems could weigh heavily on national healthcare costs and need to be addressed by healthcare professionals [12]. Therefore, the care of critically ill patients does not end upon ICU discharge [13]. Transition of care is associated to medical errors [14], the risk for readmission [15], morbidity [16], and increased mortality [17]. Thus, integrated and ongoing care post-ICU discharge is pivotal to reduce the emotional impact of ICU admission.

Person-centered care, which is based on patient values and outcome driven health care, is an important foundation for the provision of integrated healthcare [18, 19]. This person-centered approach triggers a shift from traditional provider-centric norms to care that addresses the individual beliefs and needs [20]. It includes elements such as respecting a person’s values and preferences, empathy, and communication, which may improve the overall quality of patient care [21, 22]. This health service research project focused on the emotional support of patients and relatives during and after discharge from the ICU as an essential part of person-centered care.

Ideally, aftercare starts during ICU admission. However, continued emotional support after discharge from the ICU is frequently lacking or is insufficient from the perspective of former ICU patients and their relatives [7, 23, 24]. This gap is mostly due to an insufficient knowledge of the possible consequences of the ICU experience in nurses, GPs, and society [25, 26]. The number of ICU admissions can be expected to increase with changing demographics, increasing comorbidities, and further improvements to ICU care. Due to this rising number, long-term outcomes become increasingly important. Thus, there is a need to develop interventions that reduce the emotional impact of the ICU experience for patients and their relatives, thereby improving the assessment of the quality of care as provided by ICU professionals. Both optimal emotional support and the aftercare of patients and their relatives, as part of a person-centered care approach in the ICU, are predominantly addressed within the nursing work domain.

This study focused on the development of a person-centered intervention to minimize physical, cognitive and emotional consequences after discharge from the ICU. Moving towards a person-centered approach requires a challenging shift in the mindset of the healthcare professionals, which is still a work in progress [27, 28]. Possible explanations may include the difficulty of changing the professionals´ behavior and prevailing practices, a non-systematic approach for transforming problems into tailored solutions, and insufficient or intuitively chosen intervention strategies [28, 29]. Therefore, in this project, an intervention mapping (IM) framework was used to ensure that the developed intervention was based on theory and important changeable determinants to facilitate successful adoption and execution by ICU professionals [30]. To overcome these problems, this study aimed to provide insights into (determinants of) ICU discharge problems and their underlying causes in daily practice, and to systematically develop an intervention using IM to improve the quality of care.

## Methods

### Study design

The design of the current study was qualitative, as the first aim of this project was to explore determinants of poor ICU discharge outcomes. IM is an iterative six-step method, designed to systematically develop an intervention with explicit user involvement. This method was originally used in health promotion programs ranging from HIV prevention, to overweight management, to physical activity motivation [30]. The applied steps, which have been slightly modified to relate the process to the quality improvement domain [29], are presented in detail in Additional file 1. The current article mainly covers steps one to four, containing the developmental process of the intervention. A successive article will describe steps five and six, which include the program implementation and evaluation.

In IM step 1, factors related to poor ICU discharge outcomes and previously used discharge strategies were identified by reviewing literature, conducting semi-structured interviews with former ICU patients and their relatives, holding qualitative roundtable meetings with the nursing staff, and gathering advice from key informants from several hospital organizations. Findings from the problem analysis were then compiled into a PRECEDE (an acronym for Predisposing, Reinforcing and Enabling Constructs in Educational Diagnosis and Evaluation)-based logic model [31]. In IM step 2, relevant and changeable determinants of behavior were identified to compose a matrix of performance and change objectives for the nursing staff. Subsequently, in IM step 3, theory-based methods and practical applications were selected to achieve behavioral change among the nursing staff and to allow them to overcome barriers when the intervention was used. Finally the intervention was designed in IM step 4, taking into account existing interventions.

### Study population

Former ICU patients and their relatives were approached through the Foundation of Family and patient Centered Intensive Care (FCIC), which acts as a nation-wide Dutch peer support group. Six volunteers responded to a formal request to participate in the study. The qualitative roundtable meetings were conducted in two ICUs at a university medical center in the Netherlands, including a mix of neurological, neurosurgical, transplantation, medical, and trauma surgery patients. In addition, hospital wards that commonly admitted these types of patients after transfer from the ICU were also involved in the study. All nurses working in these wards (i.e., 135 specialized nurses in the mixed ICUs and 105 general ward nurses) were eligible to participate. Key informants were identified by professional acquaintance: three senior ICU nurses/scientists, a senior ICU nurse dedicated to the field of aftercare, and a social worker.

### Data collection

An exploratory literature study was performed to identify the determinants of ICU discharge problems, their underlying causes in daily practice, and current discharge interventions or strategies. To identify the available publications on discharge practice in intensive care settings, a literature search was conducted using the electronic databases PubMed and Google scholar. Searches included terms occurring anywhere in the title and main text, with publication in an unrestricted date range and limited to the English language. The following terms were used in the search builder: “discharge,” combined with one of the following keywords: “intensive care,” “ICU,” or “critical care.” This search was completed by selecting additional publications from the reference sections of the included articles.

Semi-structured telephone interviews, lasting approximately 30 min, were conducted to learn from the experiences of former ICU patients and their relatives as it pertained to the period from ICU discharge. Six interviews were conducted until no new information emerged [32]. Given the broadly discussed and theoretically sampled storytelling from previous qualitative studies, any new data from the present study was intended to confirm general trends.

Guided by findings from the literature review and interviews, vignettes were created a priori by the study investigator (MvM) containing elements of a discharge protocol. Vignettes are short scenarios in written or pictorial form. This technique is a method that can elicit perceptions, opinions, beliefs and attitudes from responses or comments by the participants [33]. The composed vignettes were used during the roundtable meetings with nursing staff to ensure that all the meetings followed a similar mode. These meetings were conducted to gather important information on the value and feasibility of diverse relevant elements prior, during and after the discharge of an ICU patient. In general, roundtable meetings aim to explore new insights in a safe environment, are characterized by discussion between equal participants, and have been used in ICU setting [34, 35]. To increase homogeneity, registered ICU and ward nursing staff participated in separate discussions. The roundtable meetings lasted 45–60 min, were organized during work time, used a convenience sample of available nurses, and were audiotaped. One researcher (MvM) led the discussions and interviews. The vignettes and questions are presented in Table 1. Although no specific pilot of the questions and vignettes was conducted, the content and face validity were enhanced by collaborating with key informants, former patients and healthcare staff volunteers. These individuals reviewed the vignettes for clarity, use of language or other issues. No one refused to participate, and no one dropped out of the study.

**Table 1:** Vignettes and questions to perform problem analysis

Composed vignettes of the roundtable meetings with the nursing staffs		
#	Vignette elements	Reference
1	Reduction of non-essential monitoring and nursing care in ICU prior to discharge.	[24, 39, 41]
	Informal preparation of the patient prior to discharge; tailored discussion of decreased monitoring, less staff, reassurance of worries.	[26, 54]
	Consultative ICU nurse, currently providing one or more consecutive evaluations of the former ICU patient, and signed off when deemed stable.	[55, 63, 64]
2	New folder with information about the differences between ICU and wards, topics covered by the nurses in semi-structured conversation (checklist). Reassurance of worries.	[26, 41, 42]
	Relatives visiting the general ward prior to discharge.	[41]
	A personalized lay summary of the patients’ ICU stay.	[25, 43, 44]
3	Distribute the booklet ´Your recovery after ICU´ to the patient or their relatives.	[54, 65]
	Acquaintance visit of the patient to the ward prior to discharge.	[41]
4	Use of diaries; this means an active involvement of patients and relatives in identifying and meeting their own needs, and offering opportunities for reflection.	[43, 54]
	Adapted handover with emotional and psychosocial situation described, supported in electronic patient file.	[45]
5	Ward nurse visiting the patient in the ICU.	
Semi-structured interview with former ICU patients and relatives		
#	Questions	
1	What was important to you prior to discharge from the ICU?	
2	What was important during discharge and the introduction to the new ward?	
3	How did you feel in the first days after discharge from the ICU?	
4	How did you feel regarding your safety in the ICU and the general ward?	
5	Did you miss specific issues in the care after you left the ICU, and if so, please explain these?	
6	What would you like to improve, assuming an ideal situation?	

### Data analysis

Notes were made at the end of the semi-structured interviews, returned for validation to the participants, and thereafter reviewed and coded. All roundtable meetings were transcribed for verbatim analysis. Coding, categorizing, and identifying themes were applied manually to analyze the qualitative results. First, transcripts were studied for content line-by-line, and codes were noted in the margins, which created a label for each sentence. Cross-checking of the emerging labels was established with the nurse managers of the wards. Then, these labels were grouped and categorized using broader themes. Thereafter, the final remaining themes were presented to key informants (*n* = 4) to reach a consensus. To guide the reporting criteria, the COREQ checklist was used in this study [36].

## Results

### Step 1: Problem analysis

The review process started with 1061 references retrieved from the electronic databases. Thereafter, duplicate references, references only published as an abstract and non-English manuscripts were removed. The remaining references were screened by the title and abstract for relevancy. Finally, an explorative sample of 33 articles were analysed to identify factors related to poor ICU discharge outcomes and previously used discharge strategies. After the early 1990s, a wealth of literature including a meta-synthesis of user experiences of critical care discharge, examined the effect of transferring ICU patients to the follow-up ward [23, 37–41]. Some major health problems have been described, such as anxiety, insecurity, a lack of trust, the need for excessive reassurance, dependency, and loneliness [24]. Several locally used interventions described in these referred studies have been depicted as candidate elements of a discharge protocol to reduce the emotional distress experienced by ICU patients and their relatives. For example, to reduce or drop non-essential monitoring in the ICU prior to discharge [24, 39, 41]; a semi-structured oral preparation of the patient with checklist items [26, 41, 42]; providing a comprehensive information booklet [40]; visiting the general ward by relatives and/or ICU patients [41]; and writing a personalized lay summary of the medical treatment and the patient’s experiences during admission by ICU nurses [25, 43, 44]. Although most of these elements proved to be valuable on their own, an evidence-based application that fully addressed the ongoing discharge process is still needed [45].

Most important themes that emerged from the qualitative phase (both the interviews and the roundtable meetings) were providing adequate and comprehensive information on the upcoming discharge, and the knowledge gap on the Post Intensive Care Syndrome (PICS). Table 2 summarizes all the qualitative results, the verbatim quotes serve as exemplars of their associated themes and were selected based on their clarity and illustrative appropriateness.

**Table 2:** Summary of the qualitative results of former ICU patients, relatives, general ward and ICU nurses

Main theme	Units of meaning
Minimize or drop monitoring	“It would have been reassuring if monitoring was paused while my husband was still in the ICU.” (ID#r2) “If you don’t monitor the arterial catheter, then it must be removed. But it isn’t very comfortable for the patient if you need to have blood samples thereafter.” (ICU nurse) “If I expect discharge, then I examine the last Ästrup and correct what is necessary. Then the arterial line is really taken out.” (ICU nurse) “I think it is obligatory to monitor the patient during ICU admission. Therefore, I won’t drop down this because of safety reasons.” (ICU nurse)
Providing information	“It is important to inform the patient that discharge is a transition from continuous monitoring to occasional rounds and that the situation is stable enough to allow for this downsizing.” (ID#r2) “I always tell them [the ICU patients] that it is different in the general ward. A general ward nurse has to look after more than two patients, but it is suitable and safe care. Not everybody prepares the patient, I know.” (ICU nurse) “It should occur both in the ICU and in the general ward. Our care doesn’t end at the doors; we should provide structured information about what is to be expected after discharge. However, they [the general ward nurses] should be more prepared.” (ICU nurse) “A structured checklist can be a good tool to use.” (ICU nurse, general ward nurse) “The information should be provided both orally and in written form for reading at their own pace and on their own time.” (general ward nurse) “Without any monitoring, it took a little time to get used to. But more importantly, they had no idea! In the beginning, I was on Mars, and I came to Pluto thereafter.” (ID#p1) “There is little knowledge among professionals. I would have greatly benefited from an informational brochure. I was very anxious about my condition, but I couldn’t talk to anyone.” (ID#p1) “I’ve encountered so much ignorance, and I felt that I was not taken seriously. Providing more information and good communication, even a five-minute talk, could really make a difference.” (ID#r1)
Acquaintance visit	“I would have appreciated meeting some of the professionals of the next ward, just to become a little more familiar with them. The reassurance of a nurse coming to the ICU would have helped me.” (ID#p1) “Involving the relatives is a good idea if it is optional. They must not feel obliged to be present during the transition to the ward.” (ICU nurse) “I think it is a great deal but that it isn’t reality. An acquaintance visit is too impractical for all of us, even if only relatives are involved. If they need to be here during transition, the general ward should provide this hospitality at the time, whereas in the ICU, we don’t know specific details of the visiting hours in all the different follow-up wards.” (ICU nurse) “That isn’t ideal; for example, even if I come in today, I might not be working tomorrow, so it isn’t very useful then.” (general ward nurse)
Time and logistical constraints	“Hurriedly and focusing on speed, the communication was very stormy. If there had been more time and opportunity to ask questions, then we would have been less stressed in the next ward.” (ID#r1) “It goes far too quickly. I was just awoken and immediately discharged. There was barely time to prepare. I was also too ´groggy´ to listen to the information at that time.” (ID#p4) “If we could work one-on-one, then we would have enough time for emotional support.” (general ward nurse, ICU nurse) “Oh no, that is really absurd. There is no time, and it isn’t safe for the patient to have an acquaintance visit to the general ward prior to discharge. But if the relatives would like to be involved and go there, that would be useful.” (ICU nurse) “I really haven’t the time to visit the patient in the ICU prior to admission to our ward!”. (general ward nurse)
Writing a lay summary	“I had no idea what had happened, why I felt like this. I wished someone had told me, wrote down a timeline, explained what I had experienced in understandable words.” (ID#p4) “Writing a lay summary, I think, it is too subjective. I wouldn’t know how to do that, how to go beyond ´patient slept well, no pain´ and still convey medical information. What is meaningful and not legally disputable or wrong? For example, we judge delirious behavior differently than the relatives do. That is difficult to describe.” (ICU nurse) “On my first day of work, I’m too unfamiliar with the patient to that.” (ICU nurse) “That will certainly help the patient and their relatives.” (general ward nurse)
Consultative ICU nurse	“I never discussed my ICU experiences at the time. I missed that enormously, and I think it would have helped me to process my feelings, my insecurity, and my anxious thoughts.” (ID#p4) “I have noticed that the patients appreciate that you’ve come. Some general attention provides confidence in their situation.” (ICU nurse) “What I see is that we often just go by to check the physical condition. The emotional processing has not yet begun on the first day after discharge. Only after four or five days does the patient start thinking about what happened. So, that is not applicable to the consultative ICU service.” (ICU nurse) “It would be nice if the ICU nurse could come for a longer time period to talk to the patient about their experiences.” (general ward nurse)
Liaison nurse	“What I’ve missed is the feeling of enough knowledge in the general ward about the impact of an ICU admission, the understanding of my fears and anxieties. It would have been nice to talk about my emotions with an independent professional with profound knowledge of the ICU.” (ID#p2) “They [the management team] should hire a special professional just to support the relatives. This is very useful and valuable work. A lot of benefit can be gained by providing deeper emotional support for the ICU patient and their relatives.” (ICU nurse) “All this should be done by a nurse without direct patient care that day. Maybe a few dedicated nurses could work on this emotional support task.” (ICU nurse)

*ID* Ideentification, # number, *r* relative, *p* former ICU patient

Former ICU patients (*n* = 4) and relatives (*n* = 2), which were considered experts in ICU experiences, emphasized the importance of effective discharge information and supportive written materials. The patients were often unable to remember receiving any form of information or described problems with content recall. They also mentioned a lack of attention to their emotional distress. Storytelling by these experts resulted in suggestions to meet their emotional challenges. A daughter made the following statement:

“*The transition from the ICU into the high care was quite scary. My mother no longer needed full monitoring at her bedside, however, I didn’t sleep that night because of this removal. The transition to the general ward was even worse and I felt very vulnerable, really thrown into the deep end. I wished we were told about the next phase, why discharge at that time, what is the difference between ICU and the general ward, and how is the usual work flow.*”

One repeatedly recommended strategy by the experts was to reduce the knowledge gap of PICS in both the ICU and general ward nursing staff. Because of this assumed constraints, the former ICU patients experienced feelings of unsafety and loneliness. Therefore, the increase of the nurses’ knowledge on the symptoms of PICS might respond to the needs of the former ICU patients and their relatives.

**Fig. 1:**
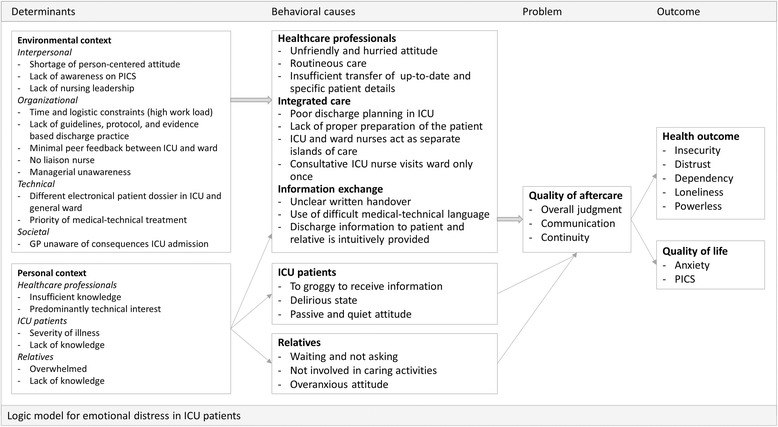
PRECEDE-based logic model adapted from Bartholomew et al. [30]

Perceptions of the various barriers and benefits of the elements in the vignettes were gleaned from the nurses’ input in the roundtable meetings (*n* = 7) (Table 3). Registered ICU (*n* = 42) and ward (*n* = 19) nursing staff (31% and 18% in respective) reported some challenges, including a decrease of patients’ comfort and concerns on their responsibility when minimizing or dropping down monitoring prior to discharge. In addition, informal patient preparation versus a structured dialogue about discharge according a checklist was brought up, in which the latter was preferred. The benefits of additional written material have been discussed, as well as barriers, such as time and logistic constraints. The nurses presented mixed feelings towards the relatives, ranging from total partnership to hesitance on involving the relatives during discharge. Writing a lay summary was met with overwhelming doubts, such as the expectation of the nurses that they had insufficient writing skills, not knowing the patient well enough, and fearing juridical consequences. The notion that a liaison nurse could provide extra support during and after discharge, was suggested spontaneously several times. Key informants recognized the findings and interpretations. Findings from the problem analysis were then compiled into a PRECEDE-based logic model [31], as shown in Fig. 1, which presents the relevant behaviors and underlying causes of the problem in a broad context.

**Table 3:** Characteristics of roundtable meeting participants (n = 61)

Roundtable	Setting	No. of participants	Female participants (%)	Discussed vignette
1	ICU	18	67	1, 2, 3
2	ICU	16	63	1, 2, 3
3	ICU	8	75	1, 2, 3
4	Neurology ward	4	100	2, 4, 5
5	Surgery ward	4	100	2, 4, 5
6	Neurosurgery ward	5	80	2, 4, 5
7	Surgery ward	6	83	2, 4, 5

### Step 2: Identify intervention outcomes, performance objectives and change objectives

Aiming to reduce emotional distress, enhancing the experience of the ICU patients’ relatives regarding discharge to the general ward was depicted as a quantifiable and feasible intervention outcome. In addition, an assessment of the overall quality of care might reflect the attitude and communication of the healthcare professionals. Attainable performance objectives for the nursing staff to facilitate transition from the ICU to the general ward are shown in Table 4. Examples of those performance objectives include ‘To prepare the ICU patient and/or the relatives to discharge according the protocol’ and ‘To listen to the concerns of the patients and their relatives’. To identify who and what should change because of the intervention, change objectives were formulated. These objectives state expected changes in behavior (e.g., introducing nurses from the general ward in the ICU) and the environment (e.g., a structured checklist regarding patient information and less monitoring prior to discharge). The new intervention should be designed to target important and changeable determinants of the nursing staff’s behavior, such as knowledge, attitudes, self-efficacy, and perceived social influence (e.g., collegial and management support) [46, 47]. A matrix of change objectives for nursing staff, based on these specified performance objectives and selected determinants, is provided in Additional file 2. This basic tool in IM describes the changes in behavioral and environmental conditions necessary to achieve the defined outcomes [30]. Examples of those outcomes include ‘Invite the relatives to be present before, during, and after discharge of the patient’ and ‘Summarize ways to value and respect a persons’ need and preferences’.

**Table 4:** Performance objectives of the nursing staff

Performance objectives	
Person-centered care	
1a	Tailor aftercare to the person’s needs and preferences
1b	Present a hospitable attitude
1c	Listen to the concerns of the patients and their relatives
1d	Involve the relatives in the discharge process
Integrated care	
2a	Improve discharge-planning in the ICU
2b	Coordinate non-technical aspects of patient care between ICU and general ward nurses
2c	Provide information on the Post Intensive Care Syndrome to the patient
Discharge communication	
3a	Prepare the ICU patient and/or relatives for discharge according the protocol
3b	Use both oral and written material in preparation for discharge
3c	Use clear language in information exchange to patients, relatives and general ward nurses

### Step 3: Select theory-based methods and practical applications

Theory-based methods related to the formulated change objectives were identified primarily from preexisting overviews [29, 30, 48–50]. A change method is a general technique for influencing the determinants of behavior and environmental conditions. These methods were then translated into practical applications that best fit the population and context for the intervention. For example, the method ‘knowledge transfer’, which was derived from the Elaboration Likelihood Method [51], bridged the nurses’ knowledge gap in PICS and suggested providing information through written material, oral explanations, and digital means (the practical application) (see Table 5). Also, the method of ‘participation’, which was derived from the Diffusion of Innovations Theory [52], was used to increase active involvement of three groups of stakeholders.

**Table 5:** Theory-based methods and practical applications

Determinant	Method (Related theory and reference)	Description (In Bartholomew et al. [30]	Examples of practical applications
Basic conditions	Participation (Diffusion of Innovations Theory [52])	Assuring high level engagement of the participants´ group in problem solving, decision making, and change activities.	Active involvement of three groups of stakeholders, using feedback of all participants, development of protocol through project group members.
	Persuasive communication (Persuasion -Communication Matrix [66], Elaboration Likelihood Method [51])	Guiding individuals and environmental agents toward the adoption of an idea, attitude, or action by using arguments or other means.	The discharge protocol is relevant, practical, and not too discrepant from the nurses’ beliefs and values.
Knowledge	Knowledge transfer (Elaboration Likelihood Method [51])	Stimulating the learner to add meaning to the information that is processed.	Bridge the nurses’ knowledge gap in PICS by providing information in written material, oral explanations, and digital means.
	Active learning (Social cognitive Theory [67])	Encouraging learning from goal-driven and activity-based experience. Need for time and information.	Group discussion on optimal discharge actions from ICU. Teacher stimulates nurses to ask questions and think of preventing PICS.
Attitude	Implementation intention (Theories of Goal Directed Behavior [68])	Prompting making if-then plans that link situational cues with responses that are effective in attaining goals or desired outcomes.	If the intended discharge becomes final, then the ICU nurse calls the contact person, starts oral conversation with the patient according to the checklist, and provides written material on PICS.
	Discussion and elaboration (Elaboration Likelihood Model [51])	Listening to arguments and opinions to ensure that the correct mental schemas are activated.	Organize team discussions on facilitators and barriers with the discharge protocol.
Self-efficacy	Skill training (Social Cognitive Theory [67])	Learning by practicing the needed skills.	Nurses feel satisfied and competent by practicing the discharge talk with an ICU patient.
	Feedback (Theories of learning [67, 69])	Giving information to nurses regarding the extent to which they are accomplishing learning.	Showing results of a pretest and posttest on PCIS.
Perceived social influence	Stimulate communication to mobilize social support (Diffusion of Innovations Theory [52], Theories of Social Networks and Social Support [70])	Combines caring, trust, openness, and acceptance with support for behavioral change, positive support is available in the environment.	Champions and nursing leaders discuss and promote performing the discharge protocol. Teachers help nurses to assimilate knowledge on PICS.
	Increasing stakeholder influence (Stakeholder theory [71])	Increase stakeholder power, legitimacy, and urgency, often by forming coalitions and using community development and social action to change an organization’s policies.	Storytelling by experts from Foundation FCIC. Patients included in focus group discussions on relevant topics

### Step 4: Develop the intervention

**Fig. 2:**
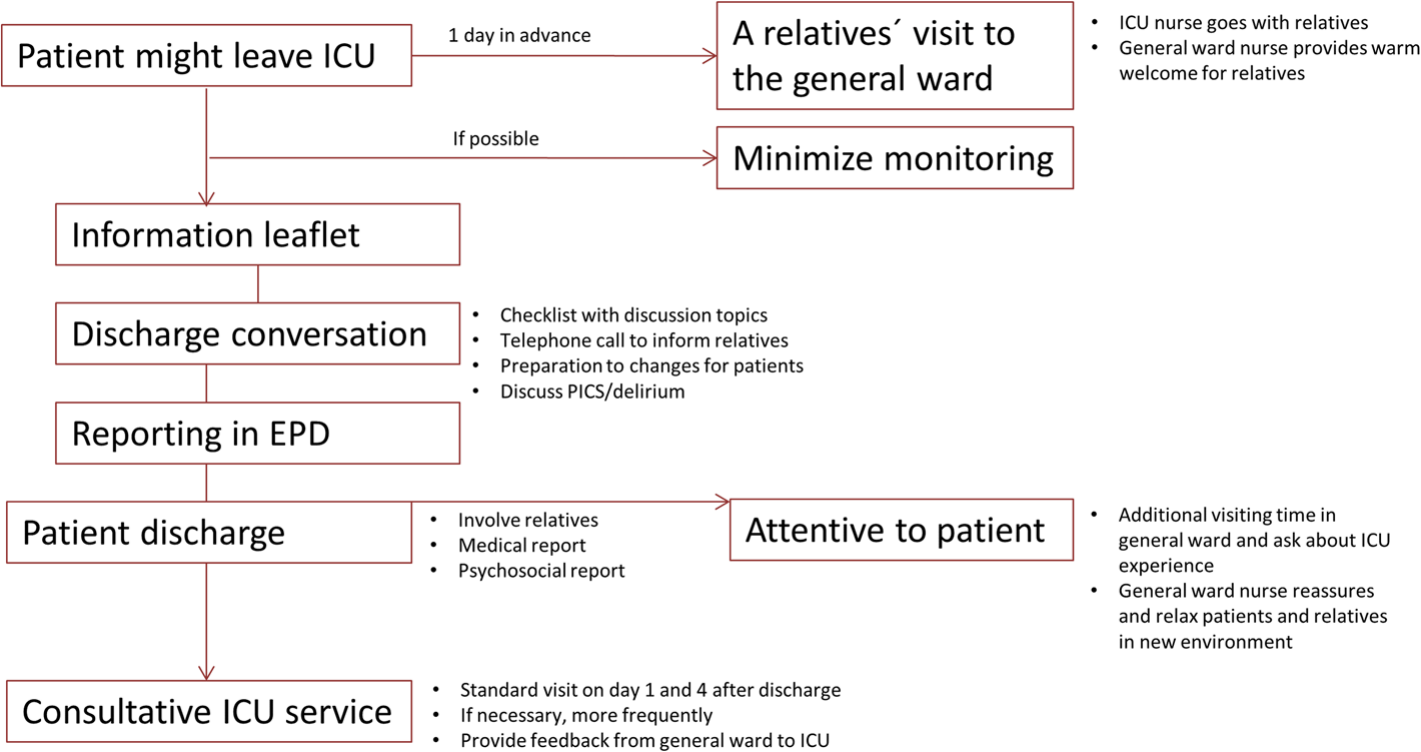
Overview of discharge protocol

Building upon the previous steps, a conceptual discharge protocol was developed that comprised valuable and achievable elements of discharge planning. It included elements currently in use in the ICUs under study, such as preparation of the ICU patient to discharge with an informational leaflet and the consultative ICU service. The protocol contained also new elements, including an acquaintance visit of the relatives to the general ward prior to discharge, and a discharge conversation with a checklist to cover the main topics to discuss. In addition, the knowledge on PCIS has been addressed. Iterative feedback from participatory work rounds with project group members from both the ICU and general wards was used to further adapt this concept to the working cultural climate in their respective units, such as extending the visiting hours immediately after the patient transfer [30]. Additional randomly selected ICU nurses, a social worker, and a former ICU patient reviewed the revised discharge protocol and provided additional input that included a provision of feedback from the general ward to the ICU. Finally, nursing managers reviewed and approved the revised discharge protocol, depicted in Fig. 2.

In addition, the knowledge on PICS has been addressed. An accompanying training module was developed to educate the ICU nurses on how to prepare and emotionally support the discharge of the patient to the general ward according the revised discharge protocol. For example, to get the patient and their relatives ready for the transfer, an oral conversation could be initiated that at least addresses the following: general information such as decreased patient monitoring, reassurance and patient safety during the discharge, physical restraints, possible emotional and cognitive consequences, and recovering from delirium or the revalidation process ahead. Educational sessions to improve the awareness of PICS in general ward nurses were established as well, ahead of further implementation of the intervention program.

## Discussion

The current research project focused on how to develop an effective person-centered discharge protocol in the ICU setting following the IM process. The problem analysis provided insights from three different user groups (experts in ICU experiences, general ward nurses, and ICU nurses). Discharge to the ward is a very personal and individual experience [23]. Like previous studies [23, 24, 39], former ICU patients and their relatives described stress-related emotions before and after ICU discharge, including anxiety, insecurity, dependency, concerns about safety in the follow-up ward, and lack of trust in their body and recovery. The North American Nursing Diagnosis Association (NANDA) defines this “relocation stress” as “a state in which an individual experiences physiological and/or psychological disturbances resulting from transfer from one environment to another” [53]. Factors related to relocation stress as part of ICU discharge may include feelings of unsafety, reduced patient monitoring, the removal of continuous nursing care because of less nursing staff, reduced information and support, delirious dreams and sleeping difficulties [24]. These factors were appointed and recognized in the roundtable meetings with nursing staff. Providing adequate and effective communication in the form of a semi-structured discharge dialogue and additional written material, has been suggested as an applicable strategy to prevent relocation stress for all stakeholders. Previous qualitative studies support these findings [24, 26, 54] and underline the need to develop an evidence based protocol to improve ICU discharge. Another strategy, mentioned in both the interviews and roundtable meetings, is to reduce the knowledge gap of PICS among the nursing staff. Increasing nurses’ understanding about PICS might in turn promote a more empathic attitude. Both could help the patient and their relatives to deal with the thoughts and feelings related to ICU discharge. The reflections of general ward nurses, which included the time pressure, insufficient information, the use of unfamiliar terminology, the need for a smoother handover processes, and uncertainty regarding the dependency of patients, were also in accordance with previous studies [41, 55].

In contrast to Bench et al., [25, 44, 56], writing a lay summary seemed far less feasible in the studied wards. Although the former ICU patients highly supported this idea, ICU nurses voiced overwhelming doubts. These doubts included the expectance having insufficient skills, not knowing the patient well enough, anticipating time constraints, fearing legal consequences, and referring to parallel diaries which were already used to reduce emotional distress in relatives. Therefore, the project group decided not to adopt and implement the lay summary of ICU stay in step 2 of the IM process. The PRECEDE-based logic model and the subsequent formulated performance objectives provided a reason to focus on interventional elements like the semi-structured discharge dialogue, the involvement of the relatives, and nurses’ knowledge gain regarding PICS.

When planning any new intervention, it is important to consider the feasibility of adoption and implementation from the start of the planning process. Although many multifaceted implementation strategies may be proposed [57], their adoption and implementation may run into problems in real world settings. An extensive review of practical implementation strategies for the behavior change of the healthcare providers in the discharge process made clear that there was a lack of evidence-based interventions that guided the professionals to improve the discharge process [29]. Regarding the ICU discharge, some new interventions have been effective; however, no guidance in change objectives of the professionals was described. Successful implementation of interventions such as the discharge protocol starts with the identification of barriers and facilitators [58, 59]. Step 1 of the IM process, the problem analysis, identified the determinants of behavior and environmental contributors that might have influenced the process of discharge from the ICU. Respect for the expertise, values, viewpoints, and contributions of all stakeholders was represented by their active participation throughout the IM developmental process. Therefore, broad support for the intervention was established from the beginning of the project. In a comprehensive review of patient discharge from the ICU, thirty patients, providers, and institutional factors in different phases of the transfer process were identified [40]. This emphasized the overall complexity of facilitators (e.g., collaboration between the ICU and the ward and the use of best practices) and barriers (e.g., provider work load, family anxiety) to successful discharge. Therefore, it is unlikely that a simple universal strategy can address the challenge of an ideal discharge from the ICU.

Involvement of the target group is essential for successful adoption, implementation, and maintenance of the revised discharge protocol [30, 57]. Therefore, ICU and general ward nurses were included in the project group from the beginning of this health service research project. Former ICU patients and their relatives were also involved as expert opinions to emphasize the views and experiences of these stakeholders. Further analysis of both barriers and facilitators to adoption and implementation of the revised discharge protocol are beyond the scope of this article. An implementation plan should be developed in accordance with this analysis, with the optimal management of activities that stimulate the use of the innovation. A detailed description of evaluation planning to assess the new discharge protocol is also beyond the scope of this article. However, the effectiveness of the revised discharge protocol could be measured through an evaluation of the experiences of former ICU patients’ relatives pertaining to discharge and of the overall quality of care. A performance indicator related to the discharge dialogue could be integrated into the electronic patient file and provided as bimonthly feedback on the wards. Pre- and posttest surveys could be used to evaluate the nurses’ knowledge gain regarding PICS. Finally, a process evaluation could be conducted to assess feasibility, generalizability and adoptability in daily nursing practice.

The strength of this study was the evidence-based and robust methodology used to provide guidance and practical change objectives to facilitate adoption, implementation, and maintenance of the revised discharge protocol. Overwhelming data demonstrated that awareness, attitudes and skills were key factors for the procedural improvement of hospital discharge [29], which was taken under advisement to establish aims and change objectives in step 2 of the IM process. As revealed in this article, the IM process helps guide program planners to access and use theory to support intervention development. Meaningful analysis of the underlying mechanisms, as illustrated in the PRECEDE-based logic model, enabled the linkage of intervention components to theory [49]. This should lead to improved outcomes for the target population and a greater potential for the replication of the intervention. Optimal supportive aftercare might enhance the quality of survivorship, and therefore promote a healthier and speedier return to society. This point of view should weaken the perceived barriers by healthcare professionals and foster a culture of partnership with patients and their relatives during and after an ICU admission.

Already from 1989, George Engel described in his Biopsychosocial model two ‘basic human needs’: 1) the need to know and to understand (e.g., interpreting physical symptoms, what can be done), and 2) the need to be known and to be understood (e.g., expressing concerns, reassurance, acceptance, and respect) [60]. Current study also found these double personal needs. In most professional-caretaker interactions both needs are addressed, however, the second need is often not expressed by the patients and their relatives and remains implicit. Therefore, affect-oriented and process-oriented communication by professionals is essential to meet this need of the caretaker. It determines the person-centeredness of the professionals and should be taken into account when developing healthcare interventions.

### Limitations

The review process was explorative, and lacked the rigorous approach of a systematic review resulting in all relevant publications in the topic of interest. Although a reference check has been done, some publications reporting successful new interventions in ICU discharge might have been missed. A difficulty in our literature search is the diversity of endpoints measured and the diversity in the groups and constructs that have been studied. On account of the comparability of results, several studies had to be excluded, reducing the total amount of quantitative evidence we could rely on. In general, published literature is the main source of evidence for making clinical and health policy decisions. By following the steps of IM, we did not limit the problem analysis to a literature review only, we completed it by performing six semi-structured telephone interviews with former ICU-patients and their relatives, and seven qualitative roundtable meetings for all eligible nurses.

One disadvantage of using the IM method, is the time-consuming aspect. Researchers in other healthcare settings that developed an intervention using IM found similar difficulties [49, 61]. Conducting a literature review, roundtable meetings, telephone interviews, project group work rounds, obtaining feedback from key informants, and the creation of matrices of change objectives, requires considerable time, effort and resources. Despite this long process, the IM method provided a theory- and empirically-based revised discharge protocol which was feasible for use in daily ICU practice, which made the time-consuming aspect worthwhile. The intervention was not as extensive as possible due to the need for simplicity and applicability.

Furthermore, this was a single-center study with limited input from former ICU patients and relatives. The results must therefore be viewed with caution and may not be applicable for broader generalization. However, the findings align with previous research on the topic. Some bias may have been introduced in the roundtable meetings and interviews, as the moderator was a former colleague at the hospital. Thus, discussion may have been influenced by the moderator’s own expectations and prejudices on the topic. However, this may also have evoked trust and confidentiality with participants, as the moderator was viewed as ´one of us´. Only four to six general ward nurses participated in each of the roundtable meetings. This might have led to a one-sided perspective on the discussed vignettes, although, the results were predominantly the same over the different roundtable meetings.

As the respondents voluntarily participated in the study, self-selection and socially desirability in their responses cannot be ruled out. Unfamiliarity with new methods, such as writing a lay summary, might have induced hesitance to embrace these methods in the discharge protocol. For this reason, the revised protocol represents a safe and practical addition into the daily work of ICU and general ward nurses.

## Conclusions

Intervention Mapping provided a comprehensive guiding framework to plan for improved ICU discharge and to facilitate use of a revised protocol. The strength of this study is the evidence-based and robust methodology used to provide guidance and practical change objectives to facilitate adoption and implementation of the intervention. Nursing ICU management may be well-versed in how to improve the discharge process to general wards, thus decreasing the emotional distress of ICU patients and their relatives.

## Additional files

Additional file 1: Intervention mapping steps, objectives and methods applied to develop the ICU discharge protocol. (DOCX 17 kb)

Additional file 2: Matrix of change objectives for the nursing staff, based on specified performance objectives and selected determinants. (DOCX 15 kb)

## Abbreviations

FCIC: Foundation Family and patient Centered Intensive Care
GP: General Practitioner
ICU: Intensive care unit
IM: Intervention mapping
N: Number
PICS: Post Intensive Care Syndrome

## References

[1] Davidson JE, Harvey MA (2016). Patient and family post–intensive care syndrome. AACN Adv. Crit. Care.

[2] Elliott D, Elliott D, Davidson JE, Harvey MA, Bemis-Dougherty A, Hopkins RO, Iwashyna TJ (2014). Exploring the scope of post–intensive care syndrome therapy and care: engagement of non–critical care providers and survivors in a second stakeholders meeting. Crit. Care Med..

[3] Griffiths JA, Morgan K, Barber VS, Young JD (2008). Study protocol: the intensive care outcome network ('ICON') study. BMC Health Serv. Res..

[4] Parker AM, Sricharoenchai T, Raparla S, Schneck KW, Bienvenu OJ, Needham DM (2015). Posttraumatic stress disorder in critical illness survivors: a metaanalysis. Crit Care Med.

[5] Harvey MA, Davidson JE (2016). Postintensive Care Syndrome: Right Care, Right Now… and Later. Crit Care Med.

[6] Bienvenu OJ, Neufeld KJ (2011). Post-traumatic stress disorder in medical settings: focus on the critically ill. Current psychiatry reports.

[7] Myhren H, Ekeberg O, Toien K, Karlsson S, Stokland O (2010). Posttraumatic stress, anxiety and depression symptoms in patients during the first year post intensive care unit discharge. Crit Care.

[8] Rattray JE, Hull AM (2008). Emotional outcome after intensive care: literature review. J Adv Nurs.

[9] Hatchett C, Langley G, Schmollgruber S (2010). Psychological sequelae following ICU admission at a level 1 academic south African hospital. South Afr J Crit Care.

[10] Pan H, Sun Y, Fu P (2009). Investigation Of anxiety and influencing factors relative to psychological needs of ICU patients' family members. Prog Mod Biomed.

[11] Needham DM, Davidson J, Cohen H, Hopkins RO, Weinert C, Wunsch H (2012). Improving long-term outcomes after discharge from intensive care unit: report from a stakeholders' conference. Crit Care Med.

[12] Cuthbertson BH, Rattray J, Campbell MK, Gager M, Roughton S, Smith A (2009). The PRaCTICaL study of nurse led, intensive care follow-up programmes for improving long term outcomes from critical illness: a pragmatic randomised controlled trial. Brit Med J.

[13] Cabrini L, Landoni G, Antonelli M, Bellomo R, Colombo S, Negro A, Pelosi P, et al. Critical care in the near future: patient-centered, beyond space and time boundaries. Minerva Anestesiol. 2015; (PMID:26474269) 26474269

[14] Horwitz LI, Moin T, Krumholz HM, Wang L, Bradley EH (2008). Consequences of inadequate sign-out for patient care. Arch Intern Med.

[15] Niven DJ, Bastos JF, Stelfox HT (2014). Critical care transition programs and the risk of readmission or death after discharge from an ICU: a systematic review and meta-analysis. Crit Care Med.

[16] Eddleston JM, White P, Guthrie E (2000). Survival, morbidity, and quality of life after discharge from intensive care. Crit Care Med.

[17] Daly K, Beale R, Chang R (2001). Reduction in mortality after inappropriate early discharge from intensive care unit: logistic regression triage model. BMJ.

[18] Barnsteiner J, Disch J, Walton M. Person and family centered care. Indianapolis, Sigma Theta Tau; 2014.

[19] Institute of Medicine (2001). Crossing the quality chasm: a new health system for the 21st century.

[20] Frampton SB, Guastello S (2014). Time to embrace a new patient-centered care rallying cry: “why not?”. Patient.

[21] Starfield BI (2011). Patient-centered care the same as person-focused care?. Perm J.

[22] Berghout M, van Exel J, Leensvaart L, Cramm JM (2015). Healthcare professionals’ views on patient-centered care in hospitals. BMC Health Serv Res.

[23] Bench S, Day T (2010). The user experience of critical care discharge: a meta-synthesis of qualitative research. Int J Nurs Stud.

[24] McKinney AA, Deeny P (2002). Leaving the intensive care unit: a phenomenological study of the patients’ experience. Int. Crit Care Nurs.

[25] Bench S, Day T, Heelas K, Hopkins P, White C, Griffiths P (2015). Evaluating the feasibility and effectiveness of a critical care discharge information pack for patients and their families: a pilot cluster randomised controlled trial. BMJ Open.

[26] Paul F, Hendry C, Cabrelli L (2004). Meeting patient and relatives’ information needs upon transfer from an intensive care unit: the development and evaluation of an information booklet. J Clin Nurs.

[27] McConnell B, Moroney T (2015). Involving relatives in ICU patient care: critical care nursing challenges. J Clin Nurs.

[28] van Mol M, Boeter G, Verharen L, Kompanje E, Bakker J, Nijkamp M. Patient-and family-centered care in the intensive care unit, A challenge in the daily practice of healthcare professionals. J Clin Nurs. 2016; 10.1111/jocn.13669. 10.1111/jocn.13669 27875001

[29] Hesselink G, Zegers M, Vernooij-Dassen M, Barach P, Kalkman C, Flink M (2014). Improving patient discharge and reducing hospital readmissions by using intervention mapping. BMC Health Serv Res.

[30] Bartholomew LK, Kok G, Markham CM. Planning health promotion programs: An intervention mapping approach. 4th ed. John Wiley and Amp; Sons Inc. 2016.

[31] Green LW, Kreuter MW (1992). CDC'S planned approach to community health as an application of PRECEED and an inspiration for PROCEED. J Health Educ.

[32] Mason M. Sample Size and Saturation in PhD Studies Using Qualitative Interviews. Forum Qualitative Social Research Sozialforschung. 2010;11(3):8.

[33] Barter C, Renold E (1999). The use of vignettes in qualitative research. Soc Res Update.

[34] American Evaluation Association Round table meetings. Retrieved from http://www.eval.org/p/cm/ld/fid=171 [Accessed 17–02-2017].

[35] Angus DC, Carlet J (2003). Surviving intensive care: a report from the 2002 Brussels roundtable. Intensive Care Med.

[36] Tong A, Sainsbury P, Craig J (2007). Consolidated criteria for reporting qualitative research (COREQ): a 32-item checklist for interviews and focus groups. Int J Qual Health Care.

[37] Bokinskie JC (1992). Family conferences: a method to diminish transfer anxiety. J Neuroscience Nurs.

[38] Jones CO, Donnell C (1994). After intensive care—what then?. Intens Crit Care Nurs.

[39] Saarmann L (1993). Transfer out of critical care: freedom or fear?. Crit Care Nurs Quart.

[40] Stelfox HT, Lane D, Boyd JM, Taylor S, Perrier L, Straus S (2015). A scoping review of patient discharge from intensive care: opportunities and tools to improve care. Chest.

[41] Whittaker J, Ball C (2000). Discharge from intensive care: a view from the ward. Intens Crit Care Nurse.

[42] Mitchell ML, Courtney M (2004). Reducing family members’ anxiety and uncertainty in illness around transfer from intensive care: an intervention study. Intens Crit Care Nurse.

[43] Bench S, Day T, Griffiths P (2013). Effectiveness of critical care discharge information in supporting early recovery from critical illness. Crit Care Nurse.

[44] Bench SD, Heelas K, White C, Griffiths P (2014). Providing critical care patients with a personalised discharge summary: a questionnaire survey and retrospective analysis exploring feasibility and effectiveness. Intens Crit Care Nurse.

[45] van Sluisveld N, Hesselink G, van der Hoeven JG, Westert G, Wollersheim H, Zegers M (2015). Improving clinical handover between intensive care unit and general ward professionals at intensive care unit discharge. Intensive Care Med.

[46] Fleuren M, Wiefferink K, Paulussen T (2004). Determinants of innovation within health care organizations. Int J Qual Health Care.

[47] Burke LE, Fair J (2003). Promoting prevention: skill sets and attributes of health care providers who deliver behavioral interventions. J Cardiovascular Nurs.

[48] Grol RP, Bosch MC, Hulscher ME, Eccles MP, Wensing M (2007). Planning and studying improvement in patient care: the use of theoretical perspectives. Milbank Q.

[49] Hurley DA, Hall AM, Currie-Murphy L, Pincus T, Kamper S, Maher C (2016). Theory-driven group-based complex intervention to support self-management of osteoarthritis and low back pain in primary care physiotherapy: protocol for a cluster randomised controlled feasibility trial (SOLAS). BMJ Open.

[50] Brug J (2007). Health education and behavior change. [Gezondheidsvoorlichting en gedragsverandering.].

[51] Petty R, Cacioppo JT (2012). Communication and persuasion: central and peripheral routes to attitude change.

[52] Rogers EM (2003). Diffusion of innovations.

[53] Carpenito-Moyet LJ (2006). Nursing diagnosis: application to clinical practice.

[54] Bench SD, Day T, Griffiths P (2011). Involving users in the development of effective critical care discharge information: a focus group study. Am J Crit Care.

[55] Athifa M, Finn J, Brearley L, Williams TA, Hay B, Laurie K (2011). A qualitative exploration of nurse's perception of critical outreach service: a before and after study. Austral Crit Care.

[56] Bench SD, Day TL, Griffiths P (2012). Developing user centred critical care discharge information to support early critical illness rehabilitation using the Medical Research Council's complex interventions framework. Intens Crit Care Nurs.

[57] Wensing M, Bosch M, Grol R (2010). Developing and selecting interventions for translating knowledge to action. Can Med Assosciation J.

[58] Grol R, Wensing M, Eccles M, Davis D. Improving patient care: the implementation of change in health care. New Jersey: John Wiley & Sons; 2013.

[59] van Achterberg T, Schoonhoven L, Grol R (2008). Nursing implementation science: how evidence-based nursing requires evidence-based implementation. J Nurs Scholarsh.

[60] Borrell-Carrió F, Suchman AL, Epstein RM (2004). The biopsychosocial model 25 years later: principles, practice, and scientific inquiry. Ann Fam Med.

[61] McEachan RR, Lawton RJ, Jackson C, Conner M, Lunt J (2008). Evidence, theory and context: using intervention mapping to develop a worksite physical activity intervention. BMC Public Health.

[62] Central Committee on Research Involving Human Subjects (CCMO). Available from: http://www.ccmo.nl/en/non-wmo-research [cited 27 April 2017].

[63] Stelfox HT, Bastos J, Niven DJ, Bagshaw SM, Turin T, Gao S (2016). Critical care transition programs and the risk of readmission or death after discharge from ICU. Intensive Care Med.

[64] Chaboyer W, Thalib L, Alcorn K, Foster M (2007). The effect of an ICU liaison nurse on patients and family's anxiety prior to transfer to the ward: an intervention study. Intens. Crit Care Nurse.

[65] ICU-steps. Intensive Care. [Een gids voor patienten en hun naasten] A guide for patients and their relatives. 2013. Available from: http://www.icusteps.org/assets/files/booklet/languages/dutch.pdf

[66] McGuire WJ, Rice R, Atkin C (2001). Input and output variables currently promising for constructing persuasive communications. Public communication campaigns.

[67] Bandura A (1986). Social foundations of thought and action: a social cognitive theory.

[68] Gollwitzer PM, Sheeran P (2006). Implementation intentions and goal achievement: a meta-analysis of effects and processes. Advances Exp Soc Psych.

[69] Kazdin AE (2012). Behavior modification in applied settings.

[70] Heaney CA, Israel BA (2008). Social networks and social support. Health behavior and health education: theory, research. Practice.

[71] Brown LD, Bammer G, Batliwala S, Kunreuther F (2003). Framing practice-research engagement for democratizing knowledge. Action Res.

