# Supplementary material for: Using an intervention mapping approach to develop a discharge protocol for intensive care patients
Source: BMC Health Serv Res. 2017 Dec 19;17:837. doi: 10.1186/s12913-017-2782-2 (PMC5737483; doi:10.1186/s12913-017-2782-2)
Supplement: Supplementary file 1 — Intervention mapping steps, objectives and methods applied to develop the ICU discharge protocol. (DOCX 17 kb) [file 12913_2017_2782_MOESM1_ESM.docx]

Additional file 1

*Intervention mapping steps, objectives and methods applied to develop the ICU discharge protocol*

| Steps | Objectives | Methods |
| --- | --- | --- |
| 1. Perform problem analysis | |  |
|  | 1.1 Establish meaningful participation of stakeholders for idea generation, decision making and product development | Composing a local project group.  Broad involvement through:  - Managerial support of the ICUs and general wards,  - Nurses’ active contribution,  - Thoughts of former ICU patients and relatives,  - Members of foundation FCIC (Family and patient Centered Intensive Care) as experts in the domain,  - Social worker,  - Reflections of scientists in the work field. |
|  | 1.2 Gain insight into target population, health problem, underlying causes and societal determinants | Analysis based on:  - Literature search,  - Roundtable meetings (n=7, 61 participants in total)*,  - Semi-structured interviews (n=6)*,  - Discussion with key contacts (n=4)*.  *Moderated by MvM, female, trained in interview skills during her Master Psychology, former ICU nurse and working as scientist during the study. |
|  | 1.3 Execute logic modelling | Researcher (MvM) completed PRECEDE model, with managerial and project group members’ feedback. |
| 2. Identify intervention outcomes, performance objectives and change objectives | |  |
|  | 2.1 State intervention outcomes | Nursing managers reflected on predefined goals and depicted the final intervention outcomes. |
|  | 2.2 Specify achievable performance objectives | Project group members reflected on the performance objectives. |
|  | 2.3 Select important and changeable determinants | Researchers (MvM, MN) indicated determinants in innovations within healthcare settings, chosen from literature search. Most important and changeable determinants were depicted with input from an implementation expert (EI). |
|  | 2.4 Develop matrices | In a combination of step 2.2 and 2.3 matrices for change objectives in nurses were established (MvM, MN, EI). |
| 3. Select theory-based methods and practical applications | |  |
|  | 3.1 Identify and select theoretical methods | Theory-based methods related to the formulated change objectives were mainly found from existing overviews. Researchers (MvM, EI) considered all eligible methods, and depicted the most relevant of these for the intervention. |
|  | 3.2 Select evidence-based interventions and design of practical applications | The chosen methods were successively interpreted within practical applications. |
|  | 3.3 Ensure that interventions and applications address change objectives |  |
| 4. Develop the intervention | |  |
|  | 4.1 Provide suggestions for developing an intervention | Researcher (MvM) executed iterative participatory work rounds with project group members to develop a revised discharge protocol. |
|  |  | Former ICU patients and relatives reflected on the revised discharge protocol and new suggestions were applied. |
|  | 4.2 Establish a revised discharge protocol | Nursing managers approved on the revised discharge protocol. |
|  | 4.3 Develop and pretest materials to establish intervention | Topic list for the semi-structured discharge talk, including:   - Less (no) monitoring of the patient, - Less caregivers available, - Reassurance and safety of discharge, - Staying with other patients in their room, - Different visiting hours, - ICU nurse visiting the patient next day, - Physical restraints and slow recovery, - Possible emotional and cognitive consequences, - Recovering from a delier, - The revalidation process ahead.   Pretest knowledge level on PICS.  Develop educational sessions with help of certified teachers.  Evaluate intervention materials with project group members. |
| 5. Develop an implementation plan | |  |
|  | 5.1 Provide suggestions for writing an implementation plan |  |
| 6. Planning for evaluation | |  |
|  | 6.1 Provide suggestions for writing an evaluation plan | - Evaluation of the experiences of former ICU patients’ relatives regarding discharge and of the overall quality of care, - an integrated performance indicator of discharge talk, - a pre- and posttest evaluating nurses’ knowledge gain on PICS, - process evaluation on the feasibility, generalizability and adoptability in daily nursing practice. |
|  |  |  |

Adapted from Bartholomew et al (2016) and Hesselink et al (2014)
