# Supplementary material for: Using an intervention mapping approach to develop a discharge protocol for intensive care patients
Source: BMC Health Serv Res. 2017 Dec 19;17:837. doi: 10.1186/s12913-017-2782-2 (PMC5737483; doi:10.1186/s12913-017-2782-2)
Supplement: Supplementary file 2 — Matrix of change objectives for the nursing staff, based on specified performance objectives and selected determinants. (DOCX 15 kb) [file 12913_2017_2782_MOESM2_ESM.docx]

Additional file 2

*Matrix of change objectives for the nursing staff, based on specified performance objectives and selected determinants*

| Determinants  Objectives | | Knowledge | Attitude | Self-efficacy | Perceived social influence |
| --- | --- | --- | --- | --- | --- |
|  |  |  |  |  |  |
| Person-centered care | |  |  |  |  |
|  | 1a | Describe most recognizable needs of (former) ICU patients and relatives | Summarize ways to value and respect a persons’ need and preferences | Express confidence in knowledge of needs and personal preferences of ICU patients and relatives | Explain the influence of colleagues on personal behavior in person-centeredness |
|  | 1b | Define a hospitable attitude and provide three examples | Argue the importance of person-centered care | Express confidence in providing hospitality and person-centered care | Discuss the perceived person-centeredness of colleagues |
|  | 1c | Recall five practical listening and communication skills | Express importance of listening to personal concerns | Express confidence in own listening and communication skills | Feel supported by colleagues in executing communication skills |
|  | 1d | Denominate the patients’ relatives as important partners in the total caring process of the patient | Invite the relatives to be present before, during, and after discharge of the patient | Express positive feelings towards relatives’ involvement in care | Debate the norms that enable or make it difficult for one to involve the relatives |
| Integrated care | |  |  |  |  |
|  | 2a | Identify optimal discharge planning | Discuss the pros and cons of optimal discharge planning | Present self-confidence in encouraging optimal discharge planning | Feel supported by nursing management discussing difficulties in optimal discharge planning |
|  | 2b | Analyze a pattern of all important non-technical aspects related to (ICU) caring | Show openness to non-technical aspects of caring | Express own ability to assess and respond to non-technical aspects in caring | Identify two colleagues who act as role-models in supporting non-technical aspects in caring |
|  | 2c | Explain symptoms, causes, and treatment of PICS | Show understanding and respect to symptoms of PICS | Express confidence in examining and managing symptoms of PICS | Show positive support to colleagues on learning of PICS |
| Discharge communication | |  |  |  |  |
|  | 3a | List the main topics of a semi-structured discharge conversation with ICU patients | Acknowledge positive effects of a checklist to structurally inform on discharge from ICU | Demonstrate confidence in executing a semi-structured discharge conversation based on a checklist | State four arguments to stimulate colleagues performing a semi-structured discharge conversation |
|  | 3b | Explain the diverse informational materials in preparation for discharge | Express a positive attitude using both oral and written informational material prior to discharge | Use personal strengths in providing the informational discharge conversation | Report the norms of colleagues using oral and written informational material prior to discharge |
|  | 3c | Discuss language use and reflect on clearness from the perspectives of general ward nurses, patients, and relatives | Express clear language while informing a patient, a relative and a general ward nurse | Express self-confidence in using clear language | Accept colleagues’ corrections when using non-clear language |
